# Novel Volumetric Sub-region Segmentation in Brain Tumors

**DOI:** 10.3389/fncom.2020.00003

**Published:** 2020-01-24

**Authors:** Subhashis Banerjee, Sushmita Mitra

**Affiliations:** ^1^Machine Intelligence Unit, Indian Statistical Institute, Kolkata, India; ^2^Department of CSE, University of Calcutta, Kolkata, India

**Keywords:** convolutional neural network, brain tumor segmentation, spatial-pooling and unpooling, conditional random field, multi-planar CNN, class imbalance

## Abstract

A novel deep learning based model called Multi-Planar Spatial Convolutional Neural Network (MPS-CNN) is proposed for effective, automated segmentation of different sub-regions *viz*. peritumoral edema (*ED*), necrotic core (*NCR*), enhancing and non-enhancing tumor core (*ET*/*NET*), from multi-modal MR images of the brain. An encoder-decoder type CNN model is designed for pixel-wise segmentation of the tumor along three anatomical planes (axial, sagittal, and coronal) at the slice level. These are then combined, by incorporating a consensus fusion strategy with a fully connected Conditional Random Field (CRF) based post-refinement, to produce the final volumetric segmentation of the tumor and its constituent sub-regions. Concepts, such as spatial-pooling and unpooling are used to preserve the spatial locations of the edge pixels, for reducing segmentation error around the boundaries. A new aggregated loss function is also developed for effectively handling data imbalance. The MPS-CNN is trained and validated on the recent Multimodal Brain Tumor Segmentation Challenge (BraTS) 2018 dataset. The Dice scores obtained for the validation set for whole tumor (*WT* :*NCR*/*NE* +*ET* +*ED*), tumor core (*TC*:*NCR*/*NET* +*ET*), and enhancing tumor (*ET*) are 0.90216, 0.87247, and 0.82445. The proposed MPS-CNN is found to perform the best (based on leaderboard scores) for *ET* and *TC* segmentation tasks, in terms of both the quantitative measures (viz. Dice and Hausdorff). In case of the *WT* segmentation it also achieved the second highest accuracy, with a score which was only 1% less than that of the best performing method.

## 1. Introduction

Gliomas (tumors of glial cells) represent 40% of tumors of the Central Nervous System, and 80% of all malignant brain tumors. The World Health Organization (WHO) grades these tumors based on the aggressiveness and infiltrative nature of their cells. Low-grade gliomas (LGG) are categorized as lowest- and intermediate-grades (WHO grades II and III), while high-grade gliomas (HGG) or glioblastoma constitute the highest-grade (WHO grade IV) (Louis et al., 2016). Diffuse LGGs are infiltrative brain neoplasms which affect different histological classes, and are called astrocytomas, oligodendrogliomas, and oligoastrocytomas (Louis et al., 2016). Although LGG patients are observed to have better survival than those with HGG, they often progress to secondary glioblastomas (GBMs) and eventual death (Li et al., 2013).

Accurate detection of tumor regions makes the job of the medical practitioner simpler, by allowing (i) appropriate measurement of tumor volume, (ii) growth monitoring of tumor in patients over time, and (iii) prognosis, with follow-up evaluation, and prediction of overall survival (OS). Based on the histological heterogeneity observed within a glioma tumor, its cells are partitioned into different sub-regions, i.e., peritumoral edema (ED), necrotic core (NCR), enhancing and non-enhancing tumor core (ET / NET) (Menze et al., 2015; Bakas et al., 2018). These sub-regions reflect important and clinically relevant information.

Magnetic Resonance Imaging (MRI) has become the standard non-invasive technique for brain tumor diagnosis, over the last few decades, due to its inherent improved soft tissue contrast (DeAngelis, 2001; Cha, 2006). MR imaging can effectively capture the intrinsic heterogeneity of gliomas using multimodal scans with varying intensity profiles. Typically four MR sequences *viz*. native *T*1-weighted (*T*1), *T*2-weighted (*T*2), post-contrast enhanced *T*1-weighted (*T*1*C*), and *T*2-weighted with FLuid-Attenuated Inversion Recovery (FLAIR), are used. The rationale behind using multiple sequences is the fact that different tumor regions are properly visible in different sequences, which again are complementary to each other; thereby rendering them as effective tools for accurately demarcating and distinguishing between different types of tumors (Banerjee et al., 2016a, 2017). Since gliomas are infiltrative, the sub-regions appear highly heterogeneous in MRI scans. Therefore, segmentation of Glioma sub-regions is considered to be one of the most challenging tasks in medical image analysis (Bakas et al., 2018).

Although manual segmentation of tumors is considered as the gold standard, it is time-consuming and prone to errors due to human fatigue. Therefore, there is a growing body of literature on computational algorithms, addressing this important task through supervised and unsupervised techniques (Menze et al., 2015; Banerjee et al., 2016b, 2018a,b; Mitra et al., 2017; Bakas et al., 2018). Development of such computer-aided tumor segmentation algorithms entails a lot of challenges due to the large spatial and structural variability among brain tumors. For example, segmenting HGG and LGG tumors with the same algorithm is a difficult proposition. It is also hard to compare any segmentation method with other existing ones, since they were often designed and validated on different private datasets. Such difficulty is due to various critical factors like (i) modalities used for the segmentation, (ii) state of the disease in which the image was taken (prior to treatment, or post-operative), (iii) type of the tumor (GBM or LGG, solid or infiltratively growing, primary or secondary), and can significantly influence the segmentation results.

Studies on tumor segmentation from brain MR images have been abundant in the literature. Here we provide a very recent literature review of the field. For extensive review on prior techniques, the reader is referred to (Bauer et al., 2013; Gordillo et al., 2013). Methodologically segmentation of tumors from brain MRI images can be broadly categorized under *generative* (Cuadra et al., 2004; Zacharaki et al., 2008; Menze et al., 2010; Banerjee et al., 2018a) and *discriminative* (Bauer et al., 2011; Zikic et al., 2012a,b; Wu et al., 2014; Menze et al., 2015; Bakas et al., 2018) family of models.

Generative methods are explicitly designed according to the anatomy and appearance of the tumor and the brain, and incorporate *a-priori* information for decision-making. Tumors can be modeled as outliers as compared to the expected shape and anatomy of the brain, as reported in references (Cuadra et al., 2004; Zacharaki et al., 2008). Menze et al. designed a generative probabilistic model for channel-specific segmentation of the tumor MRI in Menze et al. (2010). The generative approach in references (Gooya et al., 2012) first computes the spatial a-priori or “atlas” from healthy brain MRI scans. This is next modified using an expectation maximization (EM) algorithm, over a given set of patient images, to detect the most likely localization of the tumor therein. The concept of visual saliency is used in references (Banerjee et al., 2016b, 2018a; Mitra et al., 2017) for identifying tumor regions from brain MR images. This helps in automatically and quickly isolating the tumor region to be subsequently used for delineation. However, generative models are found to not generalize appropriately on unseen data; mainly due to their simple hypothesis functions. Their dependence on *a-priori* knowledge also makes them unsuitable to applications where this is not available.

On the other hand, *discriminative models* directly learn patterns from representation in the form of image features from the underlying training data, while not depending on any *a-priori* knowledge. These models may overfit the underlying training data, but have been shown to consistently perform well over unseen data due to their complex learned hypotheses. A hierarchical fully automated approach was presented (Bauer et al., 2011) for brain tissue segmentation, using support vector machine and conditional random fields. A combination of discriminative and generative models were developed (Zikic et al., 2012a) for the segmentation of high grade gliomas into the constituent sub-regions. This approach used decision forest as the discriminative classifier, which was fed with three unique, parameterized, contextually, and spatially aware features along with probabilities generated from Gaussian mixture models (Zikic et al., 2012b). Initial probability estimates were then used with spatially non-local features and context-sensitive decision forest for the classification of each data point. Another discriminative approach (Wu et al., 2014) used superpixels extracted from multi-modal MR images, with an SVM classifier being trained with features extracted by Gabor wavelet filters. A model-aware affinity model was defined, with its output being used alongside the SVM for application of conditional random fields theory before tumor segmentation.

Recently, Convolutional Neural Networks (LeCun et al., 1998) (CNNs or ConvNets) have been shown to work impressively on image recognition or classification problems (Krizhevsky et al., 2012). ConvNets are particularly useful for data that comes in the form of multiple arrays, like a color image. ConvNets essentially revolutionized the field of computer vision and have since become the de-facto standard for various object detection and recognition tasks (Farabet et al., 2013; Goodfellow et al., 2013; Sermanet et al., 2013; Simonyan and Zisserman, 2014). Inspired by their success, several medical imaging researchers have applied them toward abnormality detection and segmentation; particularly, for brain MRIs. 3D ConvNets were used as a voxel wise classifier (Urban et al., 2014). Instead of looking at each slice of each sequence, the 3D ConvNet works directly with the volumetric MRI sequences; classifying each voxel into tumor or background. The problems with this approach are the high computational cost incurred during training and testing phases, as well as the requirement of huge datasets. A similar approach was used (Zikic et al., 2014) with minimal pre-processing, by looking at the 3D patch around each point in the sequence and classifying the central point as one of the labels. A two-way ConvNet architecture was developed (Havaei et al., 2017) to exploit both local and global contexts of the input image. Each pixel in every 2D slice of the MRI data was classified into one of the four tumor sub-regions or background, by predicting the label of the center pixel of an *M* × *M* patch. The idea of local structure prediction was transferred (Havaei et al., 2017) to the task of predicting dense labels of pathological structures in multi-modal 3D volumes using patch-based label dictionaries. Two separate ConvNet architectures were designed (Pereira et al., 2016) for HGG and LGG-pixel wise label prediction, along with the use of small kernels of size 3 × 3 throughout the ConvNets. An ensemble of ConvNet architectures (Kamnitsas et al., 2018) was introduced for robust brain tumor segmentation. The contribution won the multimodal brain tumor segmentation challenge (BraTS) in 2017. Three popular ConvNets, such as “DeepMedic” (Kamnitsas et al., 2016), “Fully Convolutional Network (FCN)” (Long et al., 2015), and “U-Net” (Ronneberger et al., 2015) were used to generate the class-confidence of each voxel in a multimodal MRI volume, with a class having the highest confidence being assigned to be the segmentation label of that voxel.

Inspired by the success of ConvNets in brain tumor segmentation, we propose here a new deep learning method for segmentation of different sub-regions viz. ED, NCR, ET, and NET, from multi-modal MR images of the brain. An encoder-decoder type ConvNet model is designed for pixel-wise segmentation of the tumor along three anatomical planes (axial, sagittal, and coronal) at the slice level. These are then combined, using a consensus fusion strategy with a fully connected Conditional Random Field (CRF) based post-refinement (Krähenbühl and Koltun, 2011), to produce the final volumetric segmentation of the tumor and its constituent sub-regions. Novel concepts, such as spatial-pooling and unpooling (Badrinarayanan et al., 2017) are used to preserve the spatial locations of the edge pixels, for reducing segmentation error around the boundaries. A new aggregated loss function is also developed for effectively handling data imbalance.

The rest of the paper is organized as follows. Section 2 describes details of data, preparation of the patch database for ConvNet training, the proposed multi-planar Spatial-ConvNet model which uses a spatial-pooling layer, the aggregated loss function for imbalanced data handling during segmentation, and the radiomic analysis of the segmented volume of interest for overall survival prediction. Section 3 provides experimental results on the segmentation in multi-planar and multi-sequence data, with overall survival prediction. It also demonstrates their effectiveness through qualitative and quantitative analysis. Finally section 4 draws conclusions, and provides directions for future research.

## 2. Materials and Methods

In this section we present a detailed description of the brain tumor MRI dataset, and the proposed methods for tumor segmentation and patient overall survival (OS) prediction. Segmentation comprises of extraction of patches, training and testing of the segmentation model, and post-processing. The OS prediction consists of quantitative feature extraction and dimensionality reduction.

### 2.1. Dataset

**Figure 1:**
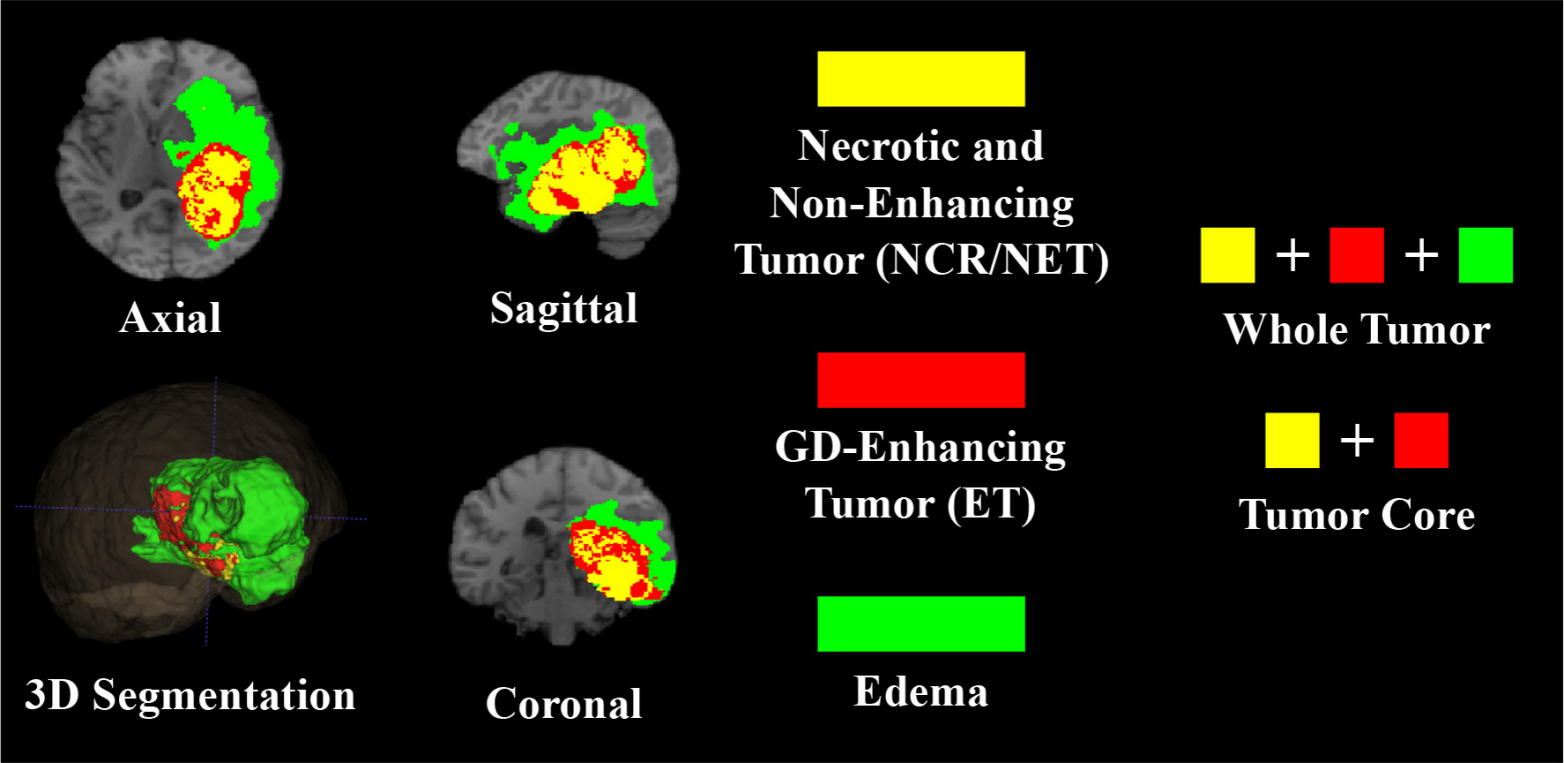
T1 MRI of a sample HGG patient with 3D segmentation of different intra-tumoral structures (ED, ET, and NCR/NET) along three principal planes (axial, sagittal, and coronal).

Multi-modal MRI volumes used in this paper, were taken from the Multimodal Brain Tumor Segmentation Challenge (BraTS) 2018^1^ (Menze et al., 2015; Bakas et al., 2017a,b,c, 2018). The dataset consists of 210 HGG and 75 LGG glioma cases as training, with 66 unlabeled (HGG or LGG) cases as validation samples. Multi-modal or multi-channel MRI volumes, consisting of *T*1, *T*1*C*, *T*2, and *FLAIR*, are available for each patient with the MRI volume being composed of 155 slices of 240 × 240 resolution. The MRI volumes are first carefully aligned to the same anatomical template, skull-stripped, and interpolated to 1mm^3^ voxel resolution, before being made available for experimentation. Manual segmentation of the tumor sub-regions is done by experts, following the same annotation protocol for all patients. Their annotations were revised and approved by board-certified neuro-radiologists. Finally, the predicted labels are evaluated by merging three regions viz. whole tumor (*WT*:*NCR*/*NE* + *ET* + *ED*), tumor core (*TC*:*NCR*/*NET* + *ET*), and enhancing tumor (*ET*) as shown in Figure 1.

### 2.2. ConvNet for Tumor Segmentation

Here we present the proposed multi planar ConvNet architecture for automatic segmentation of different tumor sub-regions, i.e., ED, ET, and NCR/NET from a given multi-modal MRI scan. Novel spatial max pooling and unpooling layers are introduced to better approximate the tumor anatomical structure by minimizing segmentation errors around the tumor boundary during up sampling. An adaptive fusion strategy for accurate and robust segmentation, by combining output from the three principal planes (axial, coronal, and sagittal), is described. A weighted aggregated loss function is introduced to train the networks in the presence of class imbalance.

#### 2.2.1. Patch Based Learning

Tumors are typically heterogeneous, depending on cancer subtypes, and contain a mixture of structural and patch-level variability. Applying a ConvNet directly to the entire slice has its inherent drawbacks. Since the size of each slice is 240 × 240, therefore overall memory requirement of the model will increase. Moreover, very little difference is observed in adjacent MRI slices at the global level; whereas, patches generated from the same slice often exhibit significant dissimilarity. We develop a Fully Convolutional Network (FCN) architecture for pixel-wise segmentation of the tumor regions. Since FCN does not contain fully connected layers, it is invariant to input image size. Therefore, we can use images of different resolutions during training and testing (or inference).

#### 2.2.2. ConvNet Architecture

The FCN architecture consists of three blocks “encoder or downsampling path,” “bottleneck,” and “decoder or upsampling path.” The encoder block contains four feature extraction blocks, each having two consecutive convolution layers with filter (or kernel) size 3 × 3. Four max-pooling layers of window size 2 × 2 are placed in between the feature extraction blocks, to down sample an image into a set of high-level features. Pairs of convolution layers are placed in the bottleneck block, between the encoder and decoder blocks. The structure of decoder block is the same as that of the encoder, with the only difference being in the use of upsampling layer instead of max-pooling to construct a pixel-wise segmentation of the input MR patch.

**Figure 2:**
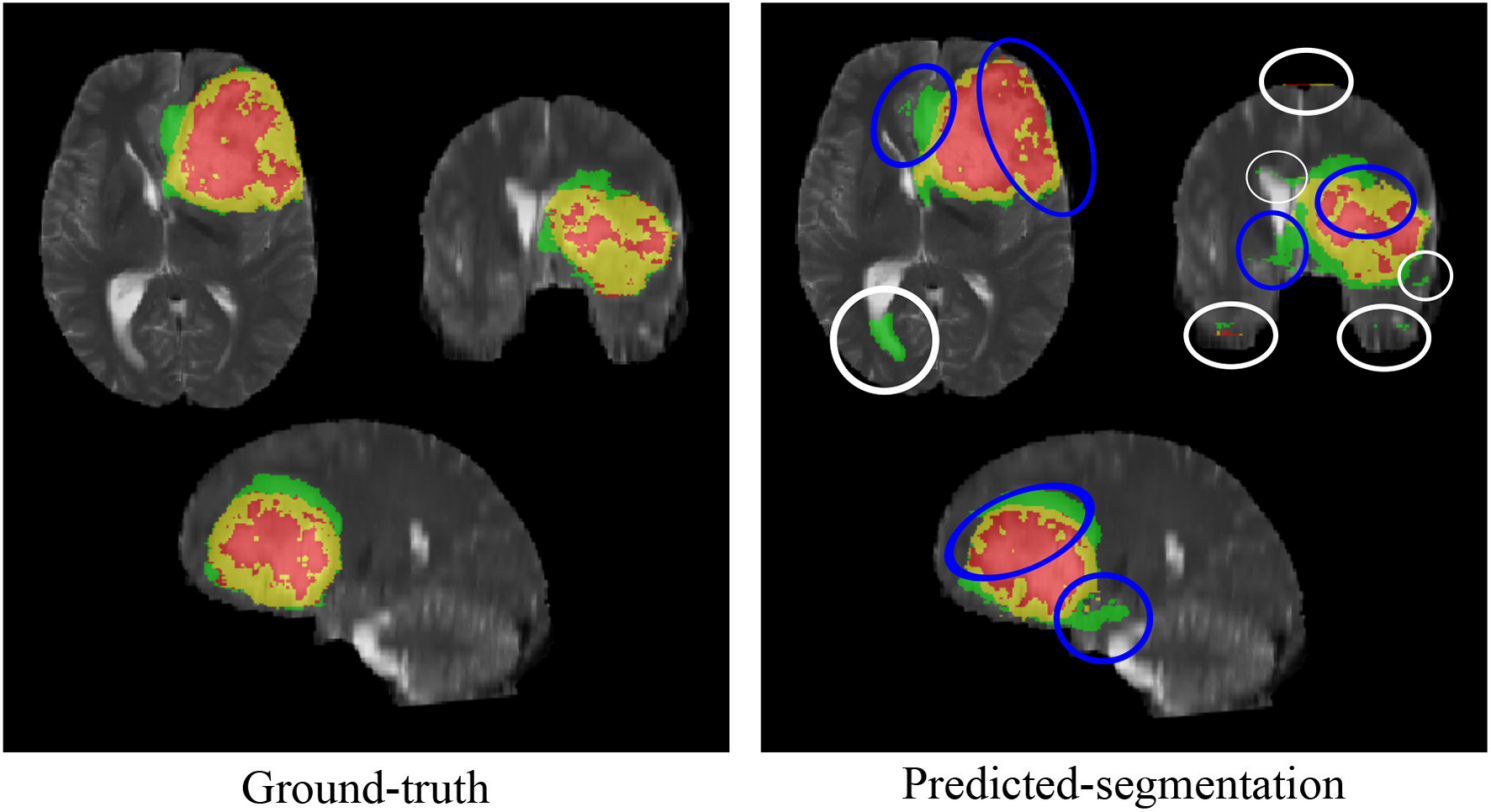
Segmentation errors, with error around the boundary marked by blue ellipse and false positive errors are marked by white ellipses.

It was observed during model validation that the predicted segmentation suffers mainly from two types of errors, as shown in Figure 2; (i) error around the boundary, and (ii) false positive at the top and bottom ends of the MRI volume. The error around the boundary occurs because the network loses spatial information during down sampling or pooling operations. The unpooling layers in the decoder block try to approximate the inverse of the pooling operation or upsample the reduced image to its original resolution through interpolation. In this process, the segmentation error percolates around the boundary of the region-of-interest (ROI) or volume-of-interest (VOI). This is considered as an important concern for a good medical image segmentation method. We name this as error around the boundary. The false positives error occur because the model is trained on 2D MRI patches without considering volumetric information.

#### 2.2.3. Spatial-Max-Pooling and Unpooling

**Figure 3:**
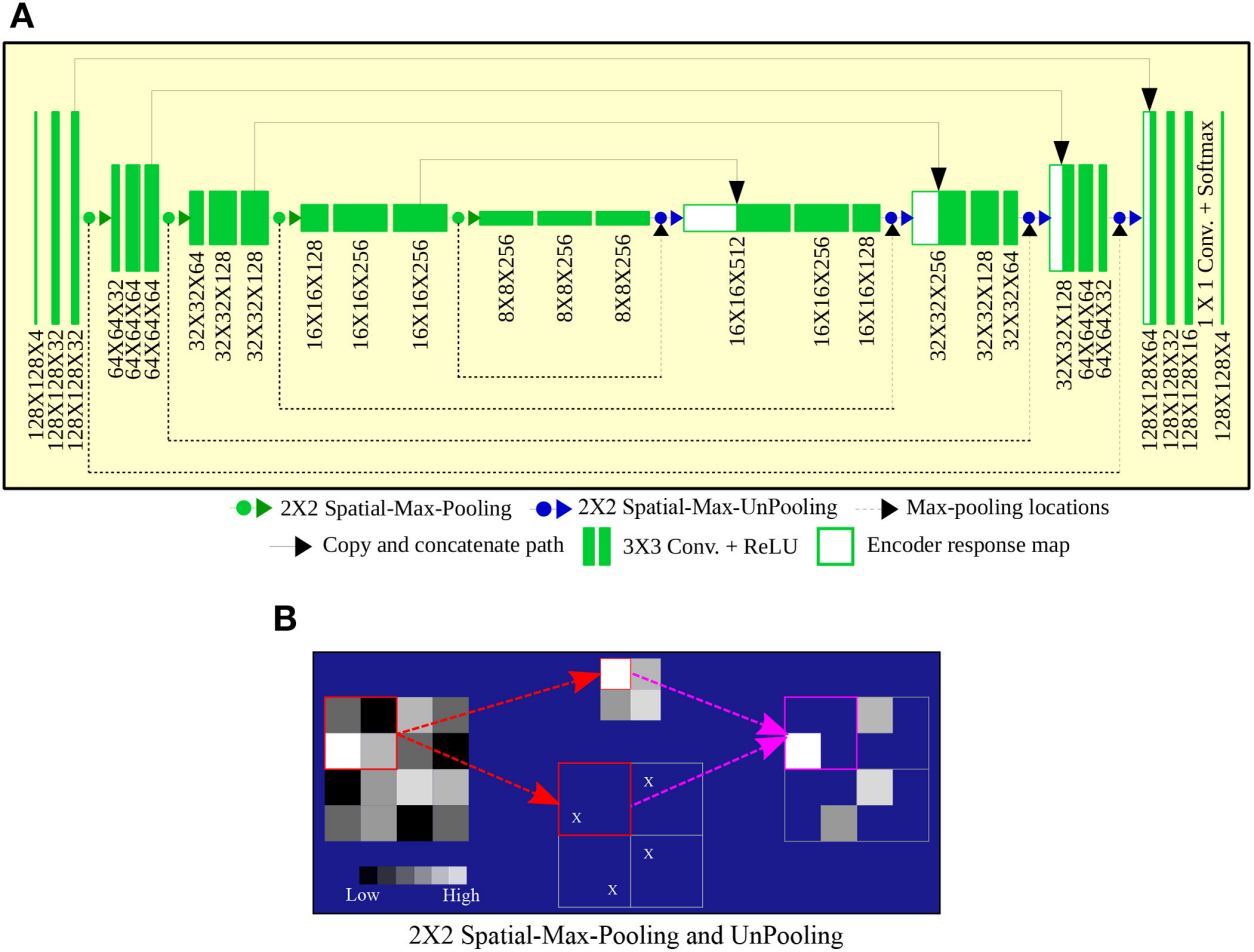
**(A)** ConvNet architecture, with **(B)** Spatial-Max-Pooling and Unpooling, for segmentation.

To circumvent the problem of error around the boundary to some extent, we used a modified version of the pooling and unpooling layers as proposed in references (Badrinarayanan et al., 2017)—and call it “spatial-max-pooling” and “spatial-max-unpooling.” Now spatial-max-pooling can retain the position from where the max-pooling operation selected the maximum value, to be subsequently used during unpooling through the spatial-max-unpooling layer. Details of the process is illustrated in Figure 3B. Although the spatial-max-pooling and unpooling layers offer an advantage over regular nearest neighbor upsampling or deconvolution, they also increase the memory requirement of the overall model. Therefore, the max pooling locations for each of the input activation maps need to be stored for a mini batch, during each such operation, and reused in subsequent mini batches. Shortcut connections are used to copy and concatenate the high resolution response maps from the encoder to the decoder. It helps the decoder network localize and recover the object details more effectively. In this way we achieve a perfect agreement between high level features and pixel level details. Figure 3A illustrates the complete architecture of the proposed ConvNet model.

#### 2.2.4. Multi-Planar Aggregation With 3D CRF Based Refinement

The MRI scans are taken in the axial (*X*-*Z*) plane, which represents voxels (or an unit volume) of the 3-Dimensional human brain. Therefore, it can be reconstructed into coronal (*Y*-*X*) plane and sagittal (*Y*-*Z*) planes for having different 3D views of the brain. Using the multi-view property of MR imaging, we propose a solution for the second error, i.e., false positive error. We train three separate ConvNets (same architecture as Figure 3A) for segmenting the tumor along the three individual planes/views. Next the predicted probability maps generated by the softmax layers of the three ConvNets (*p*_*axial*_, *p*_*coronal*_, *p*_*sagital*_) are fused by averaging the probability maps, i.e., *p* = (*p*_*axial*_ + *p*_*coronal*_ + *p*_*sagittal*_)/3. It is found that the integrated prediction from multiple planes are superior as compared to the estimated region based on any single plane in terms of accuracy, and robustness of decision. This is due to utilization of more information and minimization of the estimated loss.

Next a 3D fully-connected Conditional Random Field (CRF) based bilateral filtering (Krähenbühl and Koltun, 2011) is used to refine the fused prediction, while maintaining the local and contextual consistency of the segmentation. The 3D CRF integrates the four MRI sequences with the multi-planar fused predicted probability map, to produce an optimized segmentation by minimizing the energy function

(1) $$\begin{array}{l} E=\underset{i}{\sum }-\log p_{i}^{\left(l\right)}+\zeta \left(l_{i},l_{j}\right)\left[\omega_{1}P\left(\lambda_{i},\lambda_{j}\right)+\omega_{2}f\left(\lambda_{i},\lambda_{j}\right)\right], \end{array}$$

where

(2) $$\begin{array}{l} P\left(\lambda_{i},\lambda_{j}\right)=\exp(-\underset{d\in \left\{x,y,z\right\}}{\sum }\frac{\left|s_{i,d}-s_{j,d}\right|}{2\sigma_{\alpha ,d}^{2}}), \end{array}$$

(3) $$\begin{array}{l} f\left(\lambda_{i},\lambda_{j}\right)=\exp(-\underset{c\in \left\{T1,T1C,T2,FLAIR\right\}}{\sum }\frac{\left|I_{i,c}-I_{j,c}\right|}{2\sigma_{\gamma ,c}^{2}}-\underset{d\in \left\{x,y,z\right\}}{\sum }\frac{\left|s_{i,d}-s_{j,d}\right|}{2\sigma_{\beta ,d}^{2}}). \end{array}$$

Here $p_{i}^{\left(l\right)}$ is the fused probability of assigning label *l* to voxel *i* and ζ(*l*_*i*_, *l*_*j*_) is the label compatibility function between voxel pairs [*l*_*i*_ ≠ *l*_*j*_], with λ_*i*_ being the feature vector of voxel *i* containing seven features (viz. four intensities from the four MR sequences along with its 3D coordinate values). Note that *I*_*i,c*_ corresponds to the intensity of the *i*th voxel in the four MRI sequences denoted by *c*, and *s*_*i,d*_ represents the spatial 3D location of the voxel *i*. While function P(·) controls the smoothness of the segmented region by considering the influence of neighborhood (using the hyperparameter σ_α,*d*_), the function *f*(.) strives to preserve local and contextual consistency of the segmented output by controlling the level of similarity and proximity (using hyperparameters σ_γ,*c*_ and σ_β,*d*_). Optimizing the energy function also removes small isolated regions from the segmented output. All the model hyperparameters (α_1_, α_2_, σ_α_, σ_γ_, σ_β_) are chosen through grid searching, as reported in Table 1.

**Table 1:** Hyperparameters used for training.

**Model**	**Hyperparameters**	**Value**	
CNN	Weights and bias	Xavier (Glorot and Bengio, 2010)	
	Optimizer	ADAM (Kingma and Ba, 2014)	
	Epochs	25	
	Batch_size	16	
	Learning rate	1*e*^−^4	
	**Hyperparameters**	**Selected values**	**Values searched**
CRF	ω_1_, ω_2_	2.5, 4.0	[2, 2.5, 3, 3.5, 4], [2, 2.5, 3, 3.5, 4]
	σ_α, *x*_, σ_α, *y*_, σ_α, *y*_	24, 24, 24	[12, 24], [12, 24], [12, 24]
	σ_β, *x*_, σ_β, *y*_, σ_β, *z*_	17, 12, 10	[10−20], [10−20], [6, 8, 10, 12]
	σ_γ, *c*_	8	[4, 8, 12, 16]

**Figure 4:**
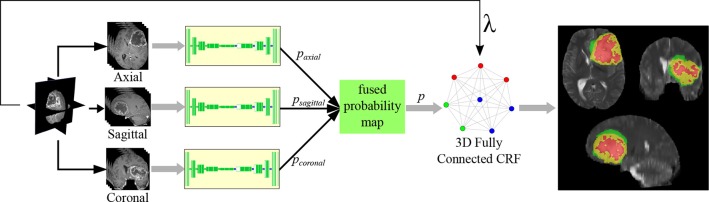
Aggregated architecture combining multiple planes, with CRF-based refinement.

The final model, represented in Figure 4, includes spatial-max-pooling and unpooling, multi-planar aggregation and 3D fully connected CRF based refinement. This will be referred to as “MPS-CNN” in the sequel.

#### 2.2.5. Loss Function for Handling Class Imbalance

**Figure 5:**
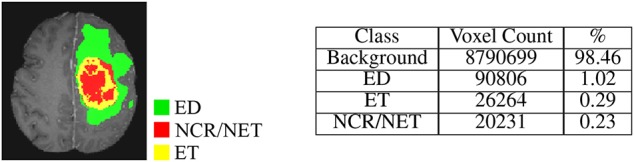
Tumor sub-class distribution for a sample MRI slice.

Since the dataset is highly imbalanced, with around 98% of the voxels belonging to either the healthy tissue or to the black surrounding area (as depicted in Figure 5), standard loss functions used in the literature are not suitable for training and optimizing the ConvNet. In such cases training can be dominated by the most prevalent class, with the classifiers focusing on learning the larger classes; thereby resulting in poor classification accuracy for the smaller classes. Therefore, we propose a new loss function. It is a sum of two factors viz.—Weighted Generalized Dice Loss (*WGDL*) (Sudre et al., 2017) and Weighted Log Loss (*WLL*) (Ronneberger et al., 2015). Both loss functions are computed between the soft binary segmentation or the probability map generated by the network using the softmax layer (*P*), and the corresponding gold standard/ground-truth image (*G*). The *WGDL* and *WLL* are defined as

(4) $$\begin{array}{l} WGDL=1-2\frac{\sum_{c=1}^{\left|C\right|}wa_{c}\sum_{n=1}^{N}G_{cn}P_{cn}}{\sum_{c=1}^{\left|C\right|}wa_{c}\sum_{n=1}^{N}G_{cn}+P_{cn}}, \end{array}$$

and

(5) $$\begin{array}{l} WLL=-\frac{1}{N}\overset{N}{\underset{n=1}{\sum }}\overset{\left|C\right|}{\underset{c=1}{\sum }}ws_{c}G_{cn}\log\left(P_{cn}\right), \end{array}$$

where *C* = {*Background, ED, ET, NCR*/*NET*}, *N* is the total number of pixels in the image. Here the contribution of each class is multiplied by the adaptive weight $wa_{c}=\frac{1}{{\left(\overset{N}{\underset{n=1}{\sum }}G_{cn}\right)}^{2}}$, which is inversely proportional to the class volume. Thereby it controls the contribution of larger classes while helping to learn smaller classes by reducing the classifier bias. Here *ws*_*c*_ is a four dimensional vector, storing the static class weights for [*Background, ED, ET, NCR*/*NET*], and is assigned based on the class ratio. Parameters *G*_*cn*_ and *P*_*cn*_ correspond to the ground truth value and the predicted output, respectively, for the *n*th pixel w.r.t. the *c*th class. Optimizing the Generalized Dice Loss (*WGDL*) produces over segmented regions, while log loss generates under-segmented regions. Therefore, we combine *WGDL* and *WLL* in a weighted fashion, so that while cross-entropy treats every pixel as an independent prediction, the dice-score looks at the resulting mask in a more holistic manner. Moreover, considering the fact that these two losses yield significantly different masks, each with its own merits and errors, a combination of such complementary information should be beneficial.

## 3. Experimental Setup and Results

The ConvNet models were developed using TensorFlow, with Keras in Python. The experiments were performed on the Intel AI DevCloud platform having cluster of Intel Xeon Scalable processors and 96 GB of RAM. The proposed segmentation model was trained and validated on the corresponding training and validation datasets provided by the BraTS 2018 (Menze et al., 2015; Bakas et al., 2017a,b,c, 2018) organizers and is described in section 2.

The CNN models were trained on the patches extracted from the standardized and cropped MRI volumes. The BraTS 2018 datasets contains MRI volumes of size 155 × 240 × 240, which are cropped to have a size of 146 × 192 × 152 for discarding some unwanted background. This helps minimize the number of patches extracted from the “non-brain” region. Then patches of size 128 × 128 (experimentally found to be the best) were extracted randomly from all the four MRI sequences, with a constraints such that the center pixel of a patch does not belong to the minimum intensity value in the *FLAIR* modality. This condition helps minimize the extraction of “non-tumor” patches. A total of 111,690, 142,160, 118,400 training patches were extracted from the axial, coronal and sagittal planes, respectively. During inference the entire stack of slices (155 × 240 × 240) of a patient is input from the test dataset, to produce pixel-wise segmentation of the tumor regions and the background.

Quantitative metrics used for evaluating the segmentation results (*P*) w.r.t. the ground truth (*G*) (in case of training) and through the Leaderboard/blind testing (in case of validation) are (i) Dice score = $\left(\frac{2|P_{1}\wedge G_{1}|}{\left|P_{1}|+|G_{1}\right|}\right)$, (ii) sensitivity = $\left(\frac{\left|P_{1}\wedge G_{1}\right|}{\left|G_{1}\right|}\right)$, (iii) specificity = $\left(\frac{\left|P_{0}\wedge G_{0}\right|}{\left|G_{0}\right|}\right)$, and (iv) Hausdorff distance = $\max\left\{\sup_{p\in \partial P1}\inf_{g\in \partial G1}d\left(p,g\right),\sup_{g\in \partial G1}\inf_{p\in \partial P1}d\left(g,t\right)\right\}$, computed for WT, TC, and ET (Menze et al., 2015). Here voxels with label 0 and 1 are denoted by *P*_0_/*T*_0_ and *P*_1_/*T*_1_, respectively. The Hausdorff distance computes maximum of the shortest least-square distance *d*, between all points on the surfaces ∂*P*1 and ∂*G*1 of the two volumes *P*1 and *G*1.

We performed two experiments to analyze (a) the effect on performance improvement through the proposed modifications in the vanilla FCN structure, and (b) the effect of the proposed aggregated loss function in terms of handling class imbalance. The hyperparameters, employed through all the experiments, are provided in Table 1. These were selected through automatic cross-validation of the baseline model. Since deep CNNs entail a large number of free trainable parameters, the effective number of training samples were artificially enhanced using real time data augmentation in the form of linear transformation like random rotation (0–10°), horizontal and vertical shifts, horizontal and vertical flips. A small part of the training set (20%) was used for validating the ConvNet model, after each training epoch, for parameter selection and detection of overfitting. Each model was trained for 20 epochs, with a single epoch consuming about an hour (approximately) on Intel AI DevCloud platform. Inference time, including 3D CRF based refinement, required about 10 min per patient (approximately).

### 3.1. Experiment 1

The proposed model MPS-CNN was compared with ten variants, as outlined below.

- **Model A**: Replacing the spatial-max-pooling and max-unpooling layers of the MPS-CNN by normal max-pooling and upsampling layers.
- **Models B–D**: Architectures same as MPS-CNN, but without incorporating multi-planar aggregation and CRF based post-processing. Models B, C, and D were trained by patches, extracted (respectively) along axial, sagittal, or coronal plane only.
- **Model E**: MPS-CNN model excluding only the CRF based post-refinement.
- **Models F–J**: Training MPS-CNN with unweighted [Equation (4) with *wa*_*c*_ = 1] and weighted dice loss (Equation 4) to generate models F and G. Next unweighted [Equation (5) with *ws*_*c*_ = 1] and weighted log loss (Equation 5) were considered to formulate models H and I. Model J was designed by training MPS-CNN with multiclass Focal loss (Lin et al., 2017), which was developed for addressing massive class imbalance.

Different models were compared based on their segmentation performance on the validation dataset, for which the organizers did not share the tumor grade (HGG/LGG) or the ground truth segmentation. During testing, the participants were required to upload the segmentation masks generated by their algorithm to the dedicated server https://www.cbica.upenn.edu/BraTS18/ for evaluation.

**Figure 6:**
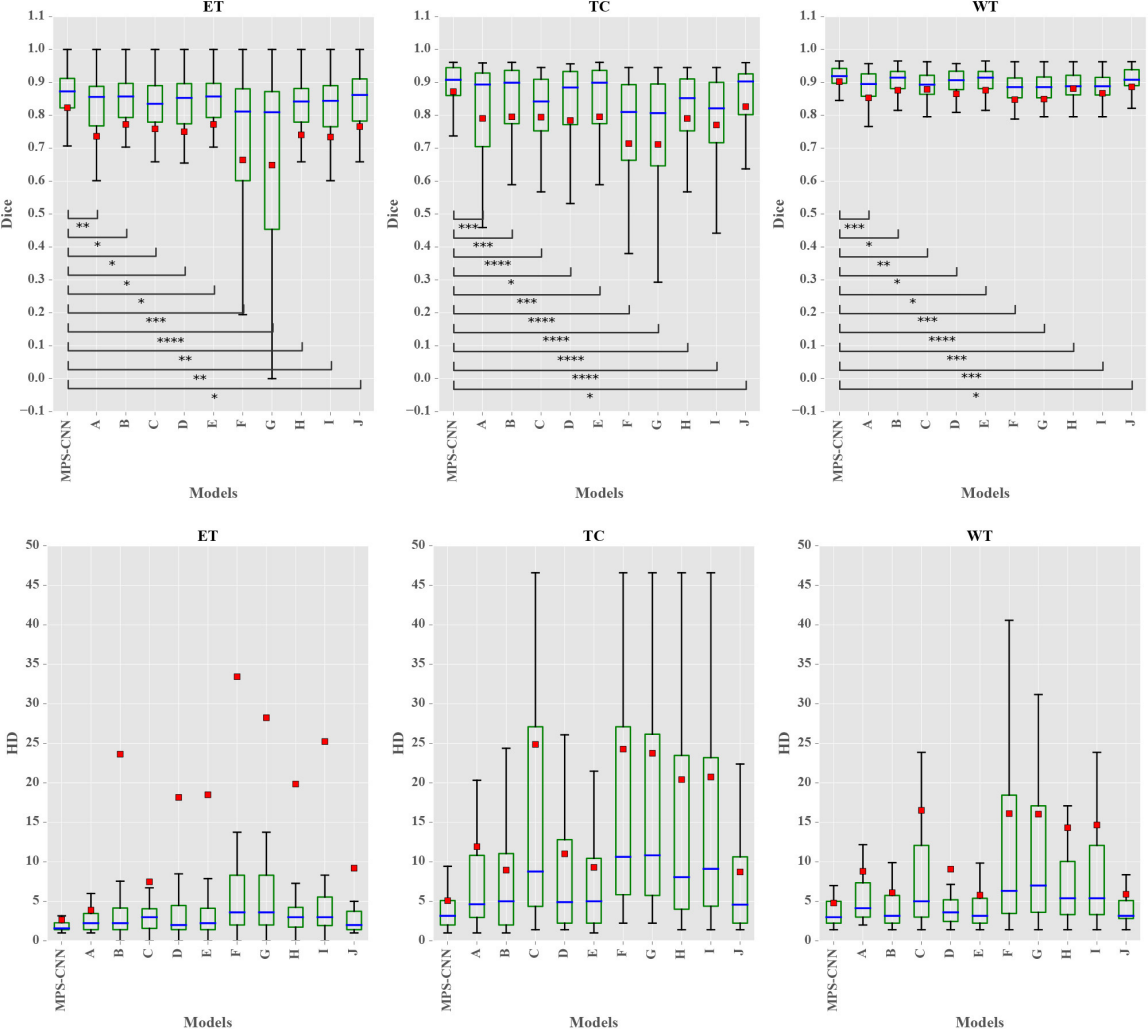
Box plots of segmentation performance for the proposed MPS-CNN and the other 10 (A–J) models, measured by Dice score and Hausdorff distance, for the WT, TC, and ET tumor sub-regions of 66 patients from the BraTS 2018 validation dataset. The *p*-values <0.05, <0.001, <0.0001, and <0.00001 for each comparison are represented by ^*^, ^**^, ^***^, and ^****^, respectively, w.r.t. MPS-CNN.

**Figure 7:**
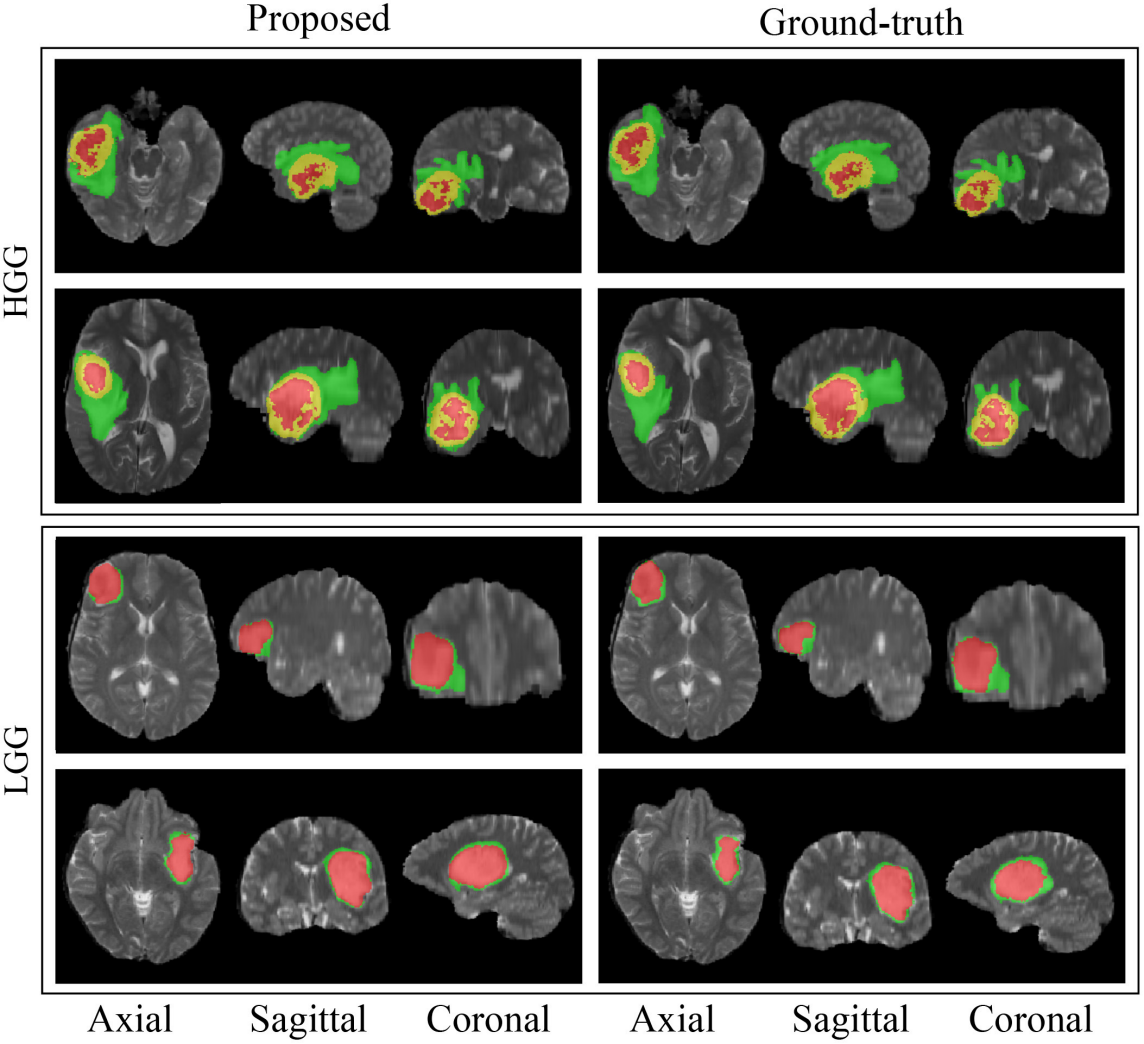
Sample segmentation results for four patients from the BraTS 2018 training dataset. The green label is edema, the red label is non-enhancing or necrotic tumor core, and the yellow label is enhancing tumor core.

The box-and-whisker plots in Figure 6 report the Dice score and Hausdroff performance of the segmentation result for the nested tumor sub-regions WT, TC, and ET for the 66 patients from BraTS 2018 validation dataset for the MPS-CNN as well as the other ten (A–J) models. The plots report the minimum & maximum; lower, median, upper quartiles; mean Dice and Hausdorff scores. The mean is marked by a red square in each case. Student's *t*-test is used to check whether the performance difference between the proposed MPS-CNN and each of the other ten compared models (A–J) is statistically significant based on their Dice score. It is evident from Figure 6 that the proposed MPS-CNN achieved the best Dice score (Dice) and Hausdorff distance (HD) for all the three tumor sub-regions (viz. ET, TC, and WT). Figure 7 demonstrates the segmentation obtained by our model MPS-CNN with reference to the corresponding ground truth, for two sample HGG and LGG patients from the training dataset.

**Figure 8:**
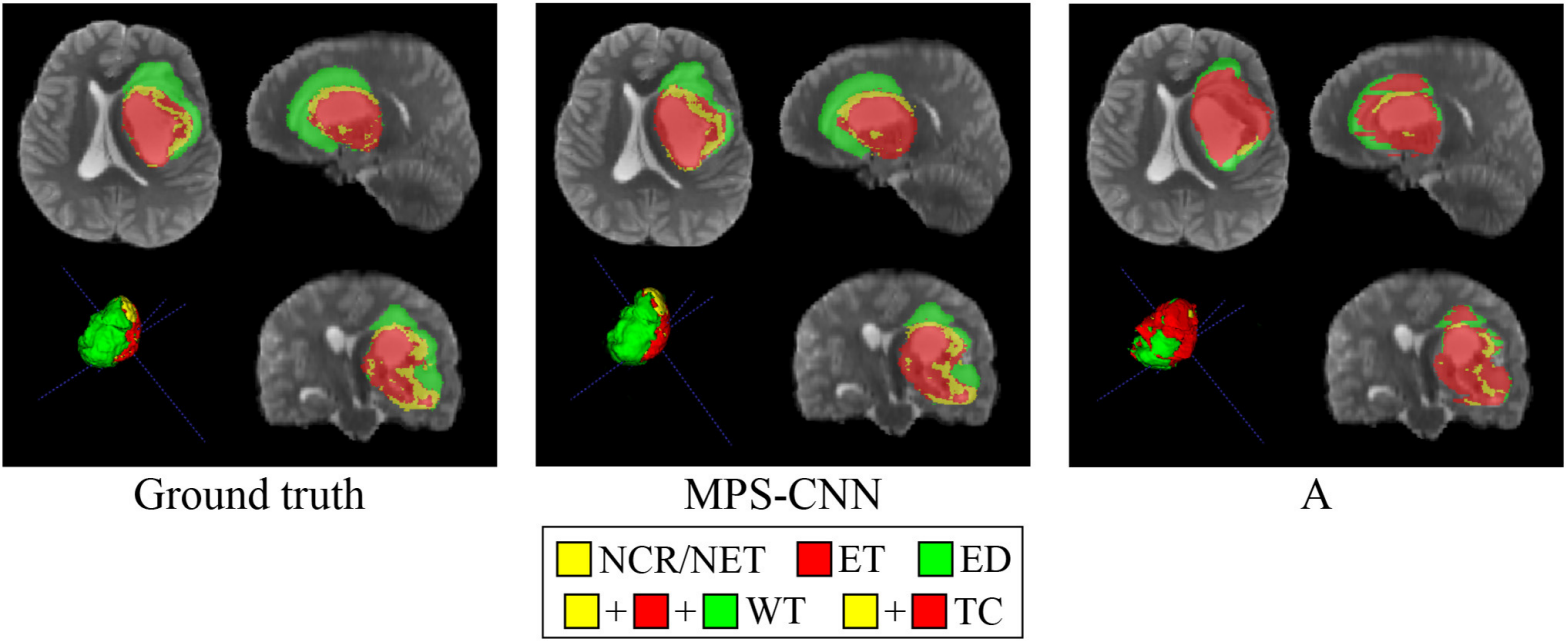
Comparative study on segmentation obtained by our model MPS-CNN, with respect to the ground truth and Model A, for a sample patient (PID: BraTS18_2013_11_1).

**Figure 9:**
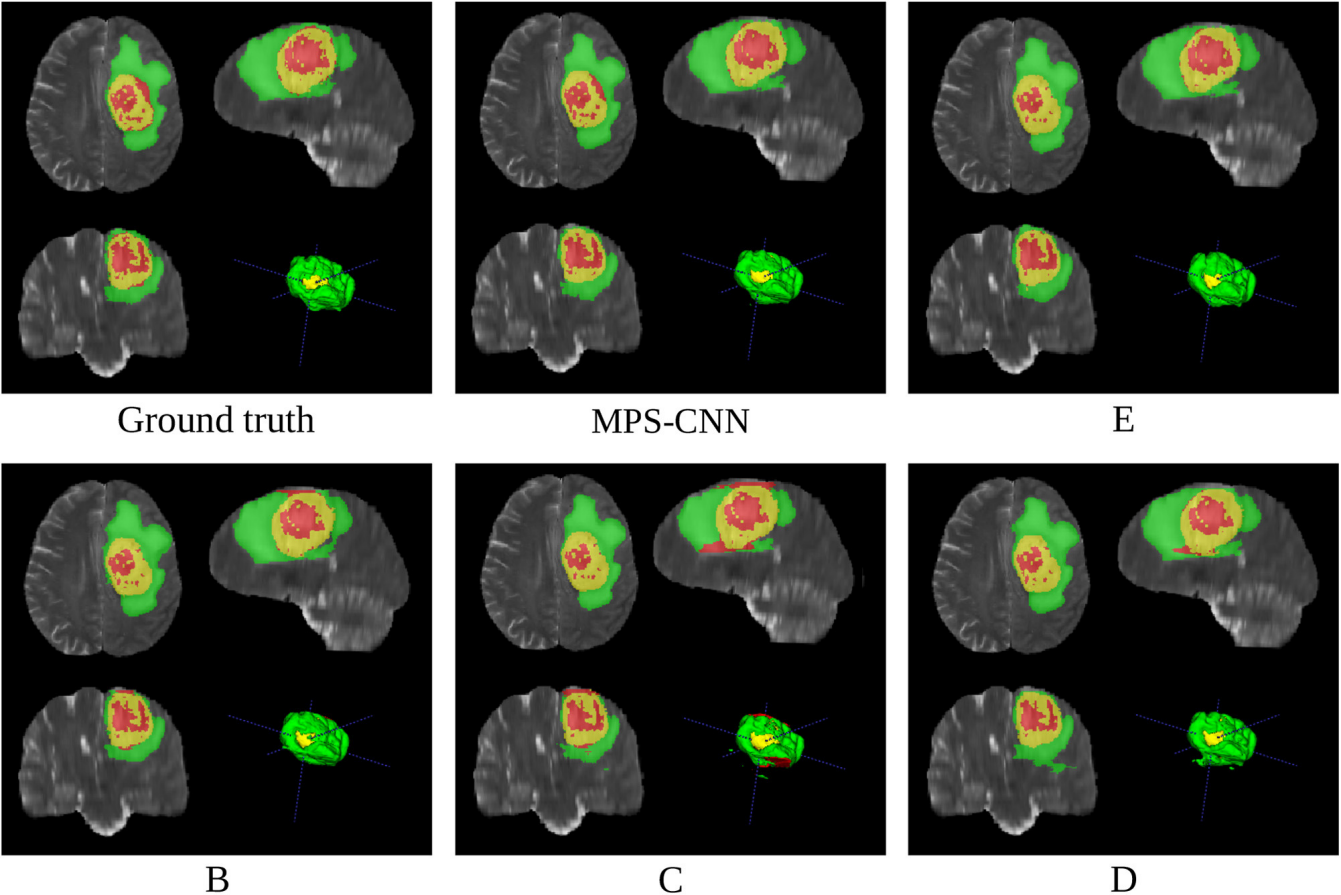
Comparative study on segmentation obtained by our model MPS-CNN, with respect to the ground truth and Models B–E, for a sample patient (PID: PID: BraTS18_2013_7_1).

Figures 8, 9 present a comparative study on the qualitative segmentation results by our model MPS-CNN and models A-E (as outlined above), to visualize the effect of the proposed modifications with respect to the basic FCN architecture. This serves to highlight the effect of the novel concepts of spatial-max-pooling and unpooling layers, along with that of multiplanar aggregation through visual demonstration on sample patients from the training dataset along all three planes (viz. axial, sagittal, coronal). Each figure also displays the ground truth segmentation. It is visually evident from Figure 9 that segmentation by model A suffers from misclassification error along the boundary of the different tumor sub-regions, with gross error in segmenting the small sub-region *ET*. On the other hand, our model MPS-CNN produced comparable segmentation w.r.t. the ground truth, for each of the tumor sub-regions.

Figure 9 demonstrates the role of multiplanar aggregation and CRF based post-processing for a sample patient. The first row presents segmentation results obtained with multiplanar aggregation with (and without) CRF based post-processing by the models MPS-CNN (and E), respectively, with reference to the corresponding ground truth. The second row illustrates segmentation by models trained on patches extracted only along a single anatomical plane (axial, sagittal, and coronal), corresponding to models B, C, D, respectively. It is clearly observed that the aggregated models, MPS-CNN and E, perform better than any of B, C, D which were trained only along a single plane. Besides, the CRF based post-processing helps MPS-CNN to achieve more structured predictions by retaining the local and contextual consistency. Thereby, some of the isolated NCR/NET regions get correctly segmented by our MPS-CNN as compared to Model E.

**Figure 10:**
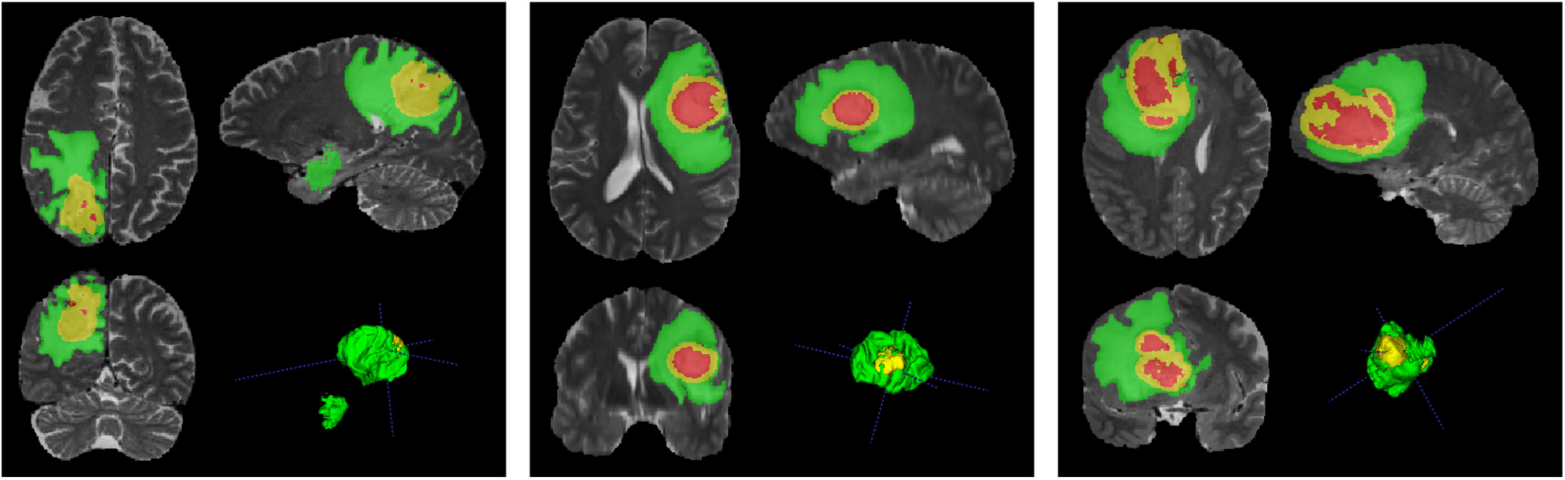
Segmentation results obtained by Model MPS-CNN on the validation dataset for three sample patients (PIDs: BraTS18_CBICA_AAM_1, BraTS18_CBICA_ALZ_1, and BraTS18_CBICA_AUE_1).

Figure 10 depicts the segmentation results, obtained by our MPS-CNN, on the validation dataset provided for three sample patients. Incidentally the models F, G, which were trained using unweighted versions of dice and log losses, were found to perform the worst due to the problem of class imbalance (as discussed in section 2.2.5). The performance gradually improved by introducing class weights to the loss functions in models H and I. However, the Focal loss function is observed to perform well in handling intra-class imbalance (for example, the amount of ET in the TC is not the same for HGG and LGG patients). However, it is less useful for cases involving inter-class imbalance.

### 3.2. Experiment 2

Our proposed model (MPS-CNN) was next compared with the top five models (based on the leaderboard performance on the validation dataset) that participated in the BraTS 2018 challenge, available online at (https://www.cbica.upenn.edu/BraTS18/lboardValidation.html). The name of our team is “radiomics-miu” and the other five teams selected for the comparison are “NVDLMED,” “SCUT_EE_CSC,” “SHealth,” “MIC-DKFZ,” and “SUSTech.” Segmentation performance of each model is measured in terms of “Dice score,” “Sensitivity,” “Specificity,” and “Hausdorff distance” (Menze et al., 2015). Three colors (red, blue, and green) are used to mark the first, second, and third highest scores, respectively (for each measure), as reported in Table 2.

**Table 2:** Comparative performance of MPS-CNN (radiomics-miu) with the top five models on the BraTS 2018 leader board (“NVDLMED,” “SCUT_EE_CSC,” “SHealth,” “MIC-DKFZ,” and “SUSTech”).

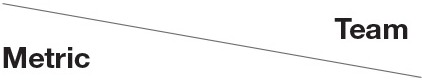		**Radiomics-miu MPS-CNN**	**NVDLMED**	**SCUT_EE_CSC**	**SHealth**	**MIC-DKFZ**	**SUSTech**
	ET	0.82445	0.82531	0.81079	0.81544	0.80871	0.80522
Dice	WT	0.90216	0.91205	0.9052	0.91204	0.91257	0.90444
	TC	0.87247	0.87049	0.85534	0.85647	0.86337	0.84943
	ET	0.86909	0.84497	0.83177	0.85053	0.83115	0.83064
Sensitivity	WT	0.91372	0.92311	0.92345	0.91968	0.91872	0.90688
	TC	0.87359	0.86405	0.87227	0.85235	0.84443	0.83156
	ET	0.99742	0.99791	0.99790	0.99753	0.99792	0.99815
Specificity	WT	0.99329	0.99519	0.99404	0.99474	0.99546	0.99549
	TC	0.99727	0.99823	0.9976	0.99773	0.99860	0.99863
	ET	2.63608	3.99705	2.5551	4.04612	2.41312	2.77719
	WT	4.74851	4.5373	4.10453	4.23619	4.26797	6.32753
Hausdorff95	TC	5.06124	6.76133	7.17313	7.21809	6.51823	6.37318

*Top three scores are marked in red, green, and blue colors, respectively*.

It is observed that our model MPS-CNN attained the highest scores in five comparisons. It performed the best for *ET* and *TC* segmentation tasks, as compared to its nearest competitor (“NVDLMED”) in terms of both the quantitative measures (Dice and Hausdorff). It is to be noted that the segmentation of *ET* and *TC* is challenging, and our MPS-CNN consistently performed best for both these tasks. In case of the *WT* segmentation it also acquired the second best accuracy, with a score which was only 1% less than that of the best performing method.

## 4. Conclusions

Manual segmentation of tumors from MRI is a highly tedious, time-consuming and error-prone task, mainly due to factors, such as human fatigue, overabundance of MRI slices per patient, and an increasing number of patients. Such manual operations often lead to inaccurate delineation. Development of automated and reproducible methodologies for accurate brain tumor segmentation is likely to have great clinical impact, since automated decision-making reduces human bias and is faster. We have developed a deep learning based model called Multi-Planar Spatial Convolutional Neural Network (MPS-CNN), for the automated segmentation of brain tumors from multi-modal MR images. The encoder-decoder type ConvNet model for pixel-wise segmentation was found to perform better than other patch-based models, mainly due to the introduction of new concepts like spatial max-pooling and unpooling to preserve the spatial locations of the edge pixels while reducing segmentation error around the boundaries. Integrated prediction from multiple anatomical planes (axial, sagittal, and coronal) was superior, in terms of accuracy and robustness of decision (as the data comes from multiple sources), with respect to the estimation based on any single plane. Shortcut connections were also incorporated to copy and concatenate the receptive fields, from the encoder to the decoder parts, to help the decoder network localize and recover the object details more efficiently. Very high segmentation scores were obtained on the test dataset in the blind testing phase. The effectiveness of the proposed aggregated loss function was demonstrated in terms of handling data imbalance, and the MPS-CNN model was found to be perform the best for the smaller classes viz. ET and TC. The CRF based post-refinement enhanced the segmentation accuracy by eliminating false positive regions.

## Data Availability Statement

Publicly available datasets were analyzed in this study. This data can be found here: https://www.med.upenn.edu/sbia/brats2018/data.html.

## Ethics Statement

The studies involving human participants were reviewed and approved by Multimodal Brain Tumor Segmentation Challenge 2018. Written informed consent to participate in this study was provided by the participants' legal guardian/next of kin.

## Author Contributions

SB conceived the experiments, conducted the experiments, analyzed the results, and wrote the manuscript with support from SM. All authors discussed the results and contributed to the final manuscript.

### Conflict of Interest

The authors declare that the research was conducted in the absence of any commercial or financial relationships that could be construed as a potential conflict of interest.

## Footnotes

^1^https://www.med.upenn.edu/sbia/brats2018/data.html
